# Chronic Effects of Coated Silver Nanoparticles on Marine Invertebrate Larvae: A Proof of Concept Study

**DOI:** 10.1371/journal.pone.0132457

**Published:** 2015-07-14

**Authors:** Christine Ying Shan Chan, Jill Man Ying Chiu

**Affiliations:** Department of Biology, Hong Kong Baptist University, Kowloon, Hong Kong; Glasgow Caledonian University, UNITED KINGDOM

## Abstract

Silver nanoparticles (AgNPs), owing to their unique physical and chemical properties, have become increasingly popular in consumer products. However, data on their potential biological effects on marine organisms, especially invertebrates, remain very limited. This proof of principle study reports the chronic sub-lethal toxicity of two coated AgNPs (oleic acid coated AgNPs and polyvinylpyrrolidone coated AgNPs) on marine benthic invertebrate larvae across three phyla (i.e., the barnacle *Balanus Amphitrite*, the slipper-limpet *Crepidula onyx*, and the polychaete *Hydroides elegans*) in terms of growth, development, and metamorphosis. Bioaccumulation and biodistribution of silver were also investigated. Larvae were also exposed to silver nitrate (AgNO_3_) in parallel to distinguish the toxic effects derived from nano-silver and the aqueous form of silver. The sub-lethal effect of chronic exposure to coated AgNPs resulted in a significant retardation in growth and development, and reduction of larval settlement rate. The larval settlement rate of *H*. *elegans* was significantly lower in the coated AgNP treatment than the AgNO_3_ treatment, suggesting that the toxicity of coated AgNPs might not be solely evoked by the release of silver ions (Ag^+^) in the test medium. The three species accumulated silver effectively from coated AgNPs as well as AgNO_3_, and coated AgNPs were observed in the vacuoles of epithelial cell in the digestive tract of *C*. *onyx*. Types of surface coatings did not affect the sub-lethal toxicity of AgNPs. This study demonstrated that coated AgNPs exerted toxic effects in a species-specific manner, and their exposure might allow bioaccumulation of silver, and affect growth, development, and settlement of marine invertebrate larvae. This study also highlighted the possibility that coated AgNPs could be taken up through diet and the toxicity of coated AgNPs might be mediated through toxic Ag^+^ as well as the novel modalities of coated AgNPs.

## Introduction

Nanoparticles (NPs) are defined as cluster of atoms with dimensions ranging between 1 to 100 nm [1]. Owing to their large surface to volume ratio, they display unique mechanical, catalytic, optical and electrical properties which can be used in a diverse range of new applications [2]. Silver nanoparticles (AgNPs) are one of the most widely used NPs in virtue of its exceptional bactericidal ability and low production cost [3]. With the extensive use of AgNPs, concerns have been raised over the impacts associated with AgNPs released into the aquatic environment via manufacturing processes or direct disposal/discharge into the landfill/aquatic system [4]. Eventually, NPs may enter the ocean either directly or via river systems [5].

The vast majority of laboratory ecotoxicological assessments on AgNPs have been conducted on the biological effects of AgNPs on freshwater species including micro-algae, arthropods, ciliophora, gastropods and fish [6–10]. Considering that the ocean is the ultimate sink of metal NPs [5], it is equally important to assess the potential impact of AgNPs on marine species. Furthermore, NP toxicity can be influenced by a variety of physiochemical properties, such as chemical composition, size, shape and coating [4]. The pH, ionic strength and solubility of the media that largely determine the size and rate of aggregation of NPs and the generation of ions of metal-based NPs may also influence its toxicity [11–13]. Therefore, AgNPs are likely to behave very differently in seawater compared to freshwater.

Recent studies have demonstrated that exposure to AgNPs could lead to mortality, developmental defects, growth inhibition and immune function and reproduction impairments of marine organisms, including diatoms, cyanobacteria, macroalgae, copepods, mussels and oysters [14–18]. For instance, it has been reported that AgNPs impaired the embryonic development of the oyster *Crassostrea virginica* and the sea urchin *Paracentrotus lividus* [19–20]. Nevertheless, while more than 80% of the marine invertebrates include a biphasic life cycle that consists of a dispersive larval stage [21], very few studies have investigated the impacts of AgNPs on the larval stage [22].

In this proof of concept study, we examined the effects of coated AgNPs on growth, development, metamorphosis and settlement of marine larvae as well as silver accumulation and particle biodistribution using three model marine invertebrate species from different phyla. The marine barnacle *Balanus amphitrite*, slipper-limpet *Crepidula onyx* and polychaete *Hydroides elegans* are major marine fouling species found worldwide that are broadly distributed in intertidal and upper sub-tidal waters. Only very limited information is available on the fate and environmental concentration of AgNPs due to the difficulties in detection and characterization of AgNPs in natural water. Therefore, it was not able to test the toxicity of AgNPs on marine invertebrate larvae using environmentally realistic concentrations. In order to investigate the mechanisms of potential toxicity of AgNPs, a range finding test was first carried out to inform the sub-lethal concentrations for use in the chronic toxicity test.

AgNPs can be produced and supplied as uncoated or coated materials. As naked AgNPs tend to aggregate which is not desirable, manufactured AgNPs are usually coated with peptides, sugars, citrate or polymers for stabilization and generating mono-dispersed suspensions that help extend and improve their unique properties. Although the coatings themselves generally do not show toxicity to organisms [23], surface coatings may play an essential role in NP toxicity and/or their mode of action [24,25]. Therefore, to better understand the importance of surface chemistry in AgNP toxicity to marine invertebrate larvae, test animals were dosed with AgNPs coated with either hydrophobic oleic acid or hydrophilic polyvinylpyrrolidone.

We also investigated whether the silver ions (Ag^+^) released were responsible for the AgNP-associated toxicity. There has been a long debate on whether AgNP-associated toxicity is mediated by the release of Ag^+^ or by the particle itself. On one hand, some studies support the theory that the toxic effect of AgNPs, whether coated or not, is derived from the release of Ag^+^ [26–28], which causes cell damage via an increase in ROS production and an induction of apoptotic pathways [29]. On the other hand, some have concluded that the AgNP effect is related to the particle itself [20,30,31]. Nevertheless, it is established that the toxicity of AgNPs is influenced by how well the particles are dispersed within the medium [10].

## Materials and Methods

### Stock preparation and characterization of coated AgNPs

TEM (particle size) and SEM (particle surface morphology) measurements were done on coated AgNPs straight from the containers they were bought in, while aggregation size characterization was performed on coated AgNPs in seawater.

Oleic acid coated AgNPs (OAgNPs) (0.2 weight % of oleic acid; nominal average particle size: 30–50 nm) and polyvinylpyrrolidone coated AgNPs (PAgNPs) (0.2 weight % of polyvinylpyrrolidone; nominal average particle size: 30–50 nm) were purchased from Nanostructured & Amorphous Materials Inc. (Los Alamos, New Mexico, USA). Particle size was determined by transmission electron microscope (TEM) (FEI Tecnai G2 20 S-TWIN, 200kV, USA). TEM photos were processed and analyzed by a public domain Java based image processing software, ImageJ (National Institute of Health, USA). The longest particle diameters were measured. Particle surface morphology was captured by scanning electron microscope (SEM) (Hitachi S-4800 FEG, Japan).

Stock solutions were prepared by dispersing 10 mg of coated AgNPs (OAgNPs or PAgNPs) in 10 mL of Milli-Q water with 0.25 mM Na_3_C_6_H_5_O_7_ for stabilization of particles [32], followed by sonication for 30 min (Sonicator B2510MTH, Branson, CT, USA). Animals showed no toxic response in this concentration of Na_3_C_6_H_5_O_7_ in preliminary study. To achieve desired concentrations for the toxicity tests (i.e. 1, 10 and 100 μg L^-1^), aliquots of the two stocks were serially diluted in membrane-filtered (pore size: 0.22 μm) natural seawater (FSW) (salinity: 33 ± 0.5 ‰; pH: 8.1 ± 0.1). The aggregation size of coated AgNPs in FSW was determined by dynamic light scattering using a Zetasizer (3000HSA, Malvern Instruments Ltd., Worcestershire, UK).

### Total dissolved silver concentration

To quantify the concentrations of silver dissolved from the two coated AgNPs in FSW, individual test solutions of 1, 10 and 100 μg L^-1^ (which were determined from preliminary range finding test; see below) were prepared in duplicate and mixed on an orbital shaker (PSU-20i, Biosan Ltd., Latvia) at 20 rpm in a 12:12 h light dark photoperiod (2.03 μmol photon m^-2^ s^-1^) at 25°C and 1 atm pressure. Low speed rotation was applied to mimic water current generated by the larval movement. The samples were analyzed for the total dissolved silver ion concentration once every 24 h. 15 mL of the solution was filtered using acid-cleaned polycarbonate membrane filter (pore size: 0.45 μm). Filtrate was centrifuged in an ultracentrifuge (Optima XL-90, Beckman Coulter, Brea, CA) at 40,000 rpm for 30 min to separate all non-soluble superfine particles, leaving only Ag^+^ at the supernatant, which was then extracted in acid digestion following Method 3005 [33]. Concentrated HNO_3_ was used instead of concentrated HCl to avoid potential silver contamination. The acidified samples were analyzed using inductively coupled plasma-optical emission spectrometer (ICP-OES) (Optima 8300, PerkinElmer, MA, USA) under conditions of 328.1 nm wavelength, 0.55 L min^-1^ neubelizer flow, 8 L min^-1^ plasma flow and 0.2 L min^-1^ auxiliary flow. Reagent blank and spike recovery samples were included to verify the analytical procedure. Three measurements were taken for each sample. The detection limit was 1.9 μg L^-1^.

### Test concentrations and range-finding test

The occurrence and environmental level of Ag have been documented since 1970s, and it has been reported that the amount of Ag dispersed into the environment could be substantial enough to pose a threat to aquatic systems [1]. However, given the limited information available on the fate and environmental concentration of AgNPs, it was not able to test the toxicity of AgNPs on marine invertebrate larvae using environmentally realistic concentrations. A range finding test was carried out to inform the sub-lethal concentrations for use in the chronic toxicity test.

Adults of *H*. *elegans*, *C*. *onyx* and *B*. *amphitrite* were collected from the low intertidal zone at Yung Shue O (22°19’N, 114°16’E), the Victoria Harbour (22°17’N, 114°10’E), and Port Shelter (22°19’N, 114°16’E) of Hong Kong, respectively. They are not endangered or protected species. No specific permissions were required for the collection of these animals at the described sites. Calcareous tubes of *H*. *elegans* were gently broken to induce spawning in Petri dishes containing membrane-filtered (pore size 0.22 μm) seawater. Eggs from five females were pooled together and mixed with sperm from only one male [34]. For *C*. *onyx*, egg capsules were located underneath the female foot and thousands of larvae were released after brooding for about two weeks [35]. Larvae of *C*. *onyx* were collected from one mother and then divided into replicates. *B*. *amphitrite* embryos in the mature egg lamellae hatched into nauplius I, which were attracted to a point-source light and subsequently, collected using a pipette [36]. Larvae of *B*. *amphitrite* were collected and pooled from multiple individuals. Larval development of this barnalce species consists of six naupliar and one cypris stages. Nauplius I is non-feeding and molts to nauplius II within 3 h of hatching.

Stage II nauplii of *B*. *amphitrite* and larvae of *H*. *elegans* were exposed to coated AgNPs at nominal concentrations of 1, 10, 100 and 1,000 μg L^-1^, while larvae of *C*. *onyx* were exposed to coated AgNPs at 100, 1,000 and 10,000 μg L^-1^. The range-finding test was conducted at 25°C (*C*. *onyx* and *H*. *elegans*) or 28°C (*B*. *amphitrite*) for 48 h in a 12:12 h light dark photoperiod.

Five replicates were set up for each larval species, coated AgNPs and concentration combination. In each replicate, twenty larvae were transferred to a beaker containing 20 mL of the test solution. The test solution was renewed and culture beakers were cleaned at 24 h. Larvae were fed on a clean algal diet of *Chaetoceros gracilis* (for *B*. *amphitrite)* or *Isochrysis galbana* (for *C*. *onyx* and *H*. *elegans*) at an initial concentration of 2 × 10^5^ cells mL^-1^ once daily [37]. To minimize algal uptake of coated AgNPs, algae were fed at least 3 h after the renewal of testing medium. Dead larvae were recorded and removed once daily. The concentration leading to 10% mortality (LC_10_) after 48 h exposure and sub-lethal concentrations for chronic toxicity test were determined. The 48 h LC_10_ value for *B*. *amphitrite*, *C*. *onyx*, and *H*. *elegans* in OAgNP treatment was 0.138, 467, and 2.63 × 10^−4^ μg L^-1^, respectively, while the 48 h LC_10_ value for *B*. *amphitrite*, *C*. *onyx*, and *H*. *elegans* in PAgNP treatment was 0.502, 38.5, and 0.317 μg L^-1^, respectively.

### Chronic toxicity test

Effects of chronic exposure for the whole larval period (i.e. 8 days for *C*. *onyx* and 5 days for *B*. *amphitrite*, and *H*. *elegans*) on growth and settlement were examined at two different sub-lethal concentrations of coated AgNPs chosen based on the results obtained from the range-finding test. Larvae of *B*. *amphitrite* and *H*. *elegans* were exposed to OAgNPs and PAgNPs at 1 and 10 μg L^-1^ (i.e. treatments O1, O10, P1 and P10), while larvae of *C*. *onyx* were exposed to higher concentrations of 10 and 100 μg L^-1^ (i.e. treatments O10, O100, P10 and P100). Negative FSW control and positive control of AgNO_3_ at concentrations of 1, 6, 11 and/or 40 μg L^-1^ were included (i.e. treatment SN1, SN6, SN11 and/or SN40). These testing concentrations of AgNO_3_ released similar dissolved silver ion concentrations as those released by the testing concentrations of coated AgNPs. SN1 corresponded to O1 and P1; SN6 to P10; SN11 to both O10 and O100; and SN40 to P100.

Each control and treatment had three (*H*. *elegans* and *C*. *onyx*) or four (*B*. *amphitrite*) replicates, and each replicate consisted of approximately 1 larva mL^-1^ in a glass beaker (capacity: 600 ml). Larvae were reared at 25°C in a 12:12 h light dark photoperiod on a clean algal diet of *I*. *galbana* (for *C*. *onyx* and *H*. *elegans*) or *C*. *gracilis* (for *B*. *amphitrite)* at 2 × 10^5^ cells mL^-1^. To minimize algal uptake of coated AgNPs, algae were fed at least 3 h after the renewal of testing medium. Testing medium was changed once daily and culture beakers were cleaned. Dead individuals were removed promptly. Approximately 10 individuals were sampled from each replicate beaker once every day, and their shell or body lengths were measured under a compound microscope (200 ×) (BX50, Olympus, Tokyo, Japan) equipped with an ocular micrometer. For *B*. *amphitrite*, another 10 to 13 larvae were sampled from each replicate to determine the developmental stage.

Settlement assays were carried out for *C*. *onyx* larvae after 8 d of exposure and for *B*. *amphitrite* and *H*. *elegans* after 5 d of exposure (*C*. *onyx* larvae were also 8 d old, and *B*. *amphitrite* and *H*. *elegans* 5 d old) following the procedures developed in our previous studies [37], in order to test if treatments would result in abnormal development of the larvae such that they could not settle and metamorphose into juveniles in response to biofilms. Briefly, biofilms were allowed to grow on Petri dishes (Falcon no. 1006) for 7 d. Petri dishes were tied to the walls of plastic baskets (L: 33 cm; W: 24 cm; H: 10 cm) using cable ties, which were deployed at the intertidal zone in the Victoria Harbour (22° 17′ 15.91″ N, 114° 10′ 25.03″ E). 10 to 15 larvae were pipetted into each replicate Petri dish containing biofilms with 10 mL filtered seawater. Clean Petri dishes served as negative control. The numbers of larvae that successfully settled on the biofilms were recorded after 6 h at 25°C (*C*. *onyx* and *H*. *elegans*) or 28°C (*B*. *amphitrite*).

### Body burden of total silver in larvae

Competent larvae were collected and pooled from each replicate beaker of FSW control, coated AgNP or AgNO_3_ treatment to obtain 10–30 mg dry weight sample for each measurement. The sample was slightly rinsed with Milli-Q water to remove any salt on the larval surface, and incubated at 65°C until constant weight was obtained. The sample was then placed in a glass vessel with 4 mL of concentrated HNO_3_ and 1 mL of 30% H_2_O_2_, digested for 2 h at 85°C and allowed to cool to room temperature. After digestion, the sample was diluted with ultrapure water to ensure ~2% HNO_3_. All digestion vessels and glassware were acid washed and rinsed with ultrapure water before use. The concentration of silver in acidified samples was quantified by inductively coupled plasma-optical emission spectrometer (ICP-OES) (Optima 8300, PerkinElmer, MA, USA) under conditions as described in section 2.2. Silver standard was purchased from PerkinElmer (Waltham, MA, USA). Calibration curve was established between 5–500 mgL^-1^ of silver standard (R^2^ value: 0.9997). Reagent blank and spike recovery samples were included to verify the analytical procedure.

### Algal uptake experiments

To characterize silver uptake through algal diet, *Chaetoceros gracilis* and *Isochrysis galbana* were first exposed to OAgNPs and PAgNP and then analyzed by ICP-OES as well as TEM. Algae were grown in f/2 medium [38] at 24°C and 33 ‰ salinity under 12:12 h light dark photoperiod. Algae were collected at stationary phase and aliquoted into conical flask. Algae were exposed to OAgNPs and PAgNPs at 1, 10 and 100 μg L^-1^ and, negative FSW control and positive control of AgNO_3_ at concentrations of 1, 6, 11 and 40 μg L^-1^ in triplicate. The exposure was conducted for 24 h at 24°C under 12:12 h light dark photoperiod. Algae were harvested onto 1 μm nitrocellulose membrane filters (Advantec) and slightly rinsed with artificial seawater (Instant Ocean synthetic seasalt; NaCl, CaCl_2_, KCl, Na_2_SO_4_, MgCl_2_; Salinity: 32 ‰; pH: 8), and dried for 24 h at 55°C. The dried samples were acid-digested and analyzed as mentioned in section 2.5.

### Biodistribution of coated AgNPs: TEM sample preparation and analysis

Stage VI nauplii and cyprids of *B*. *amphitrite*, and competent larvae of *C*. *onyx* and *H*. *elegans* were sampled from AgNO_3_ and coated AgNP treatments during the chronic toxicity test. They were fixed in 2.5% glutaraldehyde in cacodylate buffer (0.1 M sodium cacodylate-HCl buffer pH 7.4) and stored at 4°C before analysis. The larvae were washed twice with cacodylate buffer for 10 min at room temperature followed by dehydration with 50%, 70%, 90% (20 min each) and 100% ethanol (3 rinses, 20 min each). Subsequently, they were rinsed with propylene oxide for 2 times (10 min each). The larvae were infiltrated with epoxy resin and propylene oxide in 1:3 for 12 h, 1:1 for 12 h and 3:1 for 12 h followed by pure epoxy resin for 24 h at 37°C. Finally, they were embedded in epoxy resin which polymerized at 45°C overnight and then at 60°C for 2 d.

Ultra-thin sections at 100 nm thickness were stained with 2% aqueous uranyl acetate followed by TEM examination (FEI Tecnai G2 20 S-TWIN, 200kV, USA). The composition of any suspected coated AgNPs was determined using energy-dispersive X-ray spectroscopy (EDS) (Oxford Instruments, INCAx-sight EDS Detectors with INCA Energy TEM Software).

### Statistical analysis

Percentage data were arcsine transformed before analysis [39]. One-way analysis of variance (ANOVA) was used to test the difference in growth rate, settlement rate, and silver bioaccumulation of larvae and algae. If a significant difference was found by ANOVA, Tukey’s test was used for pairwise multiple comparison to identify the difference between various treatments. Data were checked for normality with Shapiro-Wilk’s *W* test [40] and for homogeneity of variance with Levene's test [39]. When either normality test or homogeneity test failed, non-parametric Kruskal-Wallis one-way ANOVA on ranks and Dunn’s test were used for analyses instead. Data were considered to be significantly different if *p* < 0.05. All statistical analyses were performed using SigmaPlot version 11.0 for Windows (Systat Software, San Jose, CA).

## Results

### Characterization of coated AgNPs

**Fig 1:**
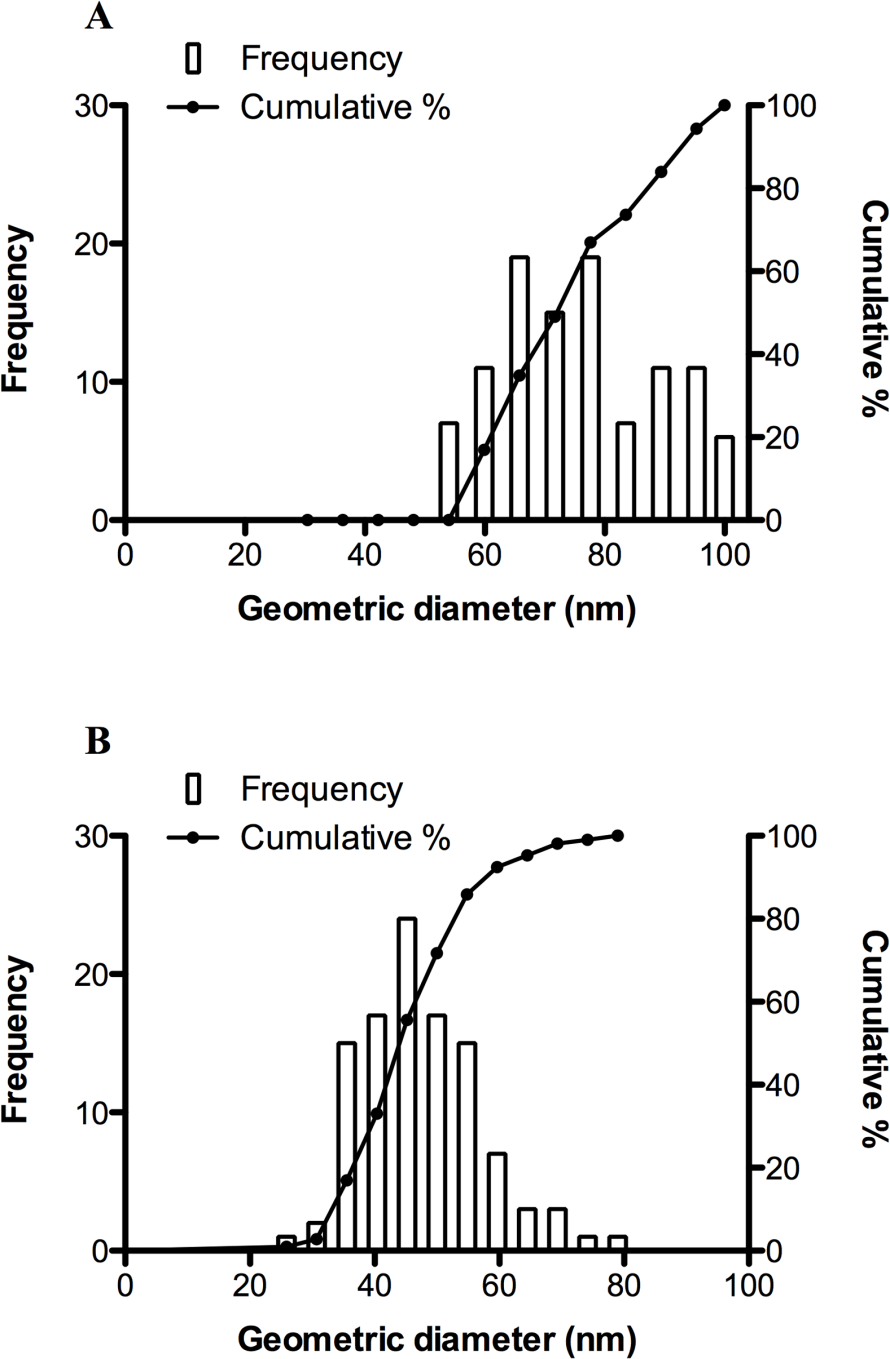
Particle size distribution by frequency percent of total particle volume and cumulative percentage of size classes. The individual graphs demonstrate the particles size of OAgNPs (A) and PAgNPs (B) counted using transmission electronic microscope (No. of particle counted = 106).

**Fig 2:**
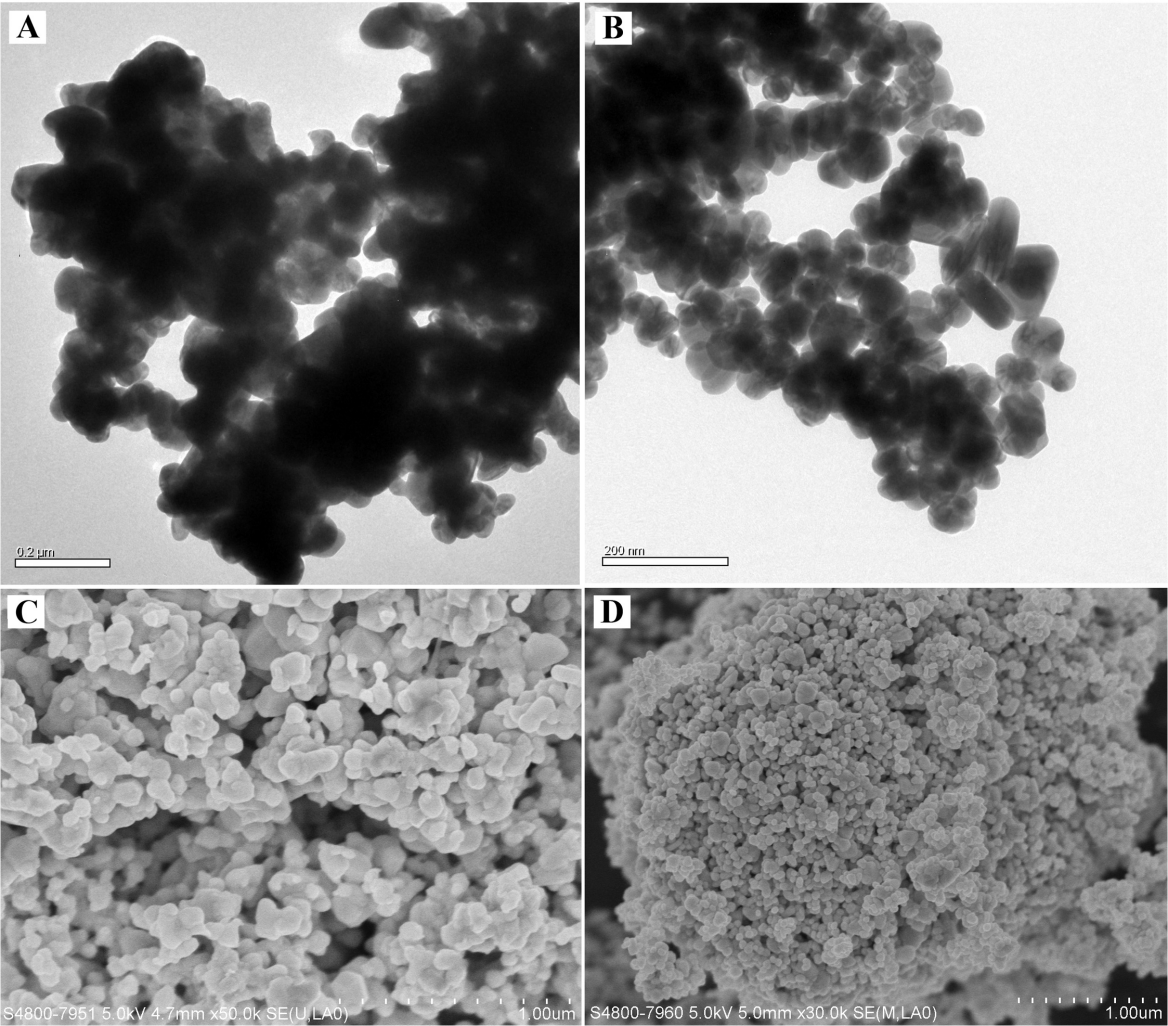
EM micrographs. Images of OAgNPs (A) and PAgNPs (B) taken by transmission electronic microscope; images of OAgNPs (C) and PAgNPs (D) taken by scanning electronic microscope. Scale bar in (A-B): 0.2 μm; (C-D): 1 μm.

The mean (± SD) particle diameter of OAgNP was 73.54 ± 1.34 nm and PAgNP was 44.86 ± 0.94 nm (Fig 1). The EM examination revealed that OAgNPs were irregular in shape and tended to bind together (Fig 2) while PAgNPs exhibited a fine singular morphology (Fig 2). The aggregation size of OAgNPs and PAgNPs no longer existed in a nanoscale in FSW. The size of aggregates increased with increasing concentration of coated AgNPs. The size of OAgNP aggregates in 1, 10 and 100 μg L^-1^ test solutions were 0.55, 1.20 and 1.72 μm, respectively. The size of PAgNP aggregates in 1, 10 and 100 μg L^-1^ test solution were 0.39, 0.62 and 0.92 μm, respectively. Furthermore, the size of OAgNP aggregates was larger than that of PAgNP aggregates at the same test concentrations.

### Total dissolved silver concentration

**Fig 3:**
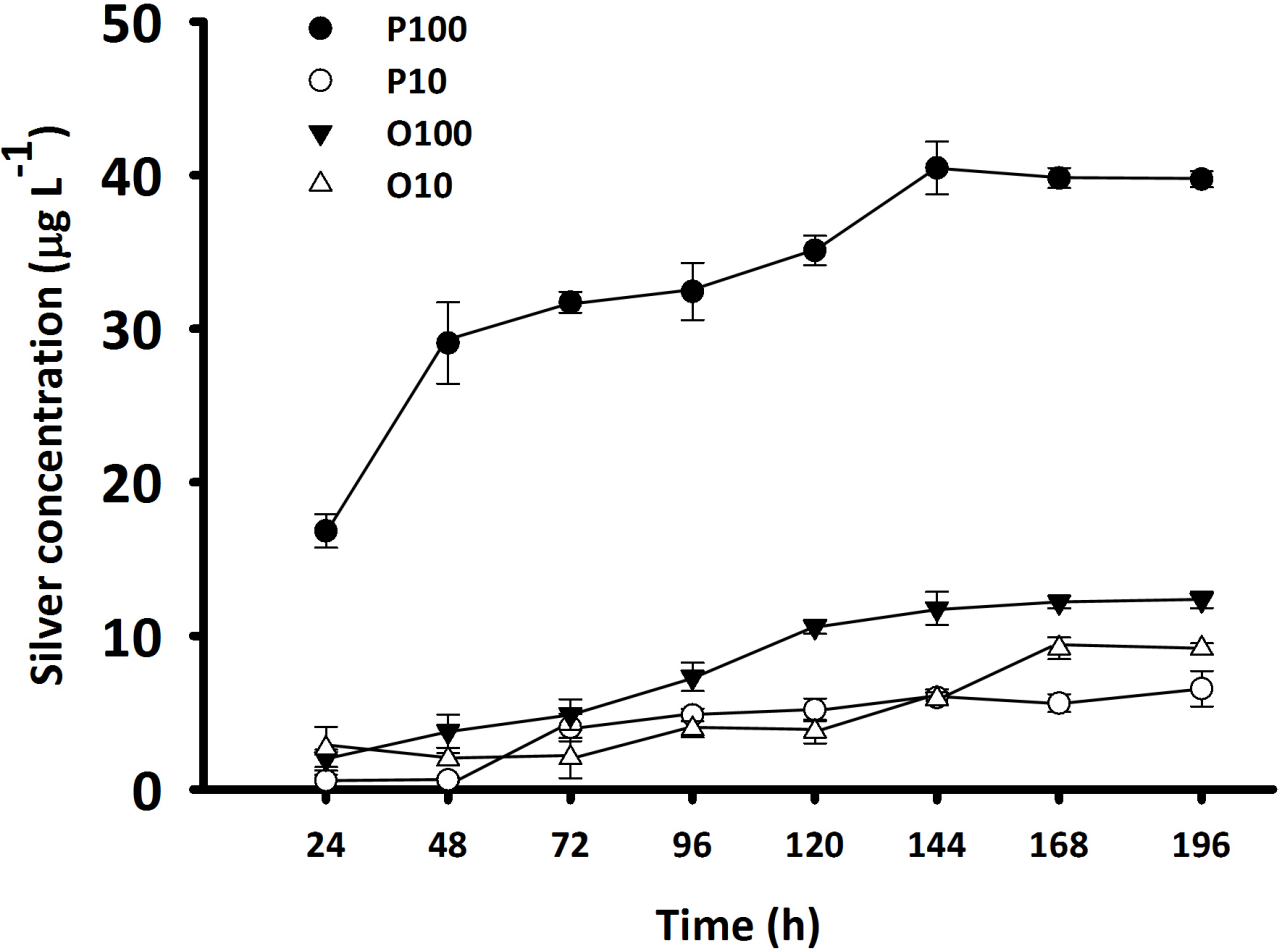
Concentration of silver released from coated AgNPs mixed in filter seawater at 10 and 100 μg L^-1^. OAgNPs at 10 μg L^**-1**^ (O10; open triangle) and 100 μg L^**-1**^ (O100; filled triangle). PAgNPs at 10 μg L^**-1**^ (P10; open circle) and 100 μg L^**-1**^ (P100; filled circle). Samples were collected every 24 h followed by ultracentrifugation for silver measurement using inductively coupled plasma-optical emission spectrometer. Each data point represents the mean (± SD) of duplicate.

The amount of Ag^+^ released from coated AgNPs increased gradually and reached equilibria after 6 d (Fig 3). At high concentrations (i.e. 100 μg L^-1^), the mean (± SD) amount of Ag^+^ released from PAgNPs (i.e. 40.00 ± 0.40 μg L^-1^) was >3 folds more than that released from OAgNPs (i.e. 12.11 ± 0.29 μg L^-1^). At low concentrations (i.e. 10 μg L^-1^), the mean (± SD) amount of Ag^+^ released from OAgNPs (i.e. 9.17 ± 0.03 μg L^-1^) was slightly higher than that released from PAgNPs (i.e. 6.05 ± 0.47 μg L^-1^). However, there were no positive readings at the lowest concentration (i.e. 1 μg L^-1^) during measurement. This might be because the amount of Ag^+^ released was below the limit of detection at 1.9 μg L^-1^.

### Sub-lethal toxicity of coated AgNPs: Larval growth, development and settlement

#### *B. amphitrite*

**Fig 4:**
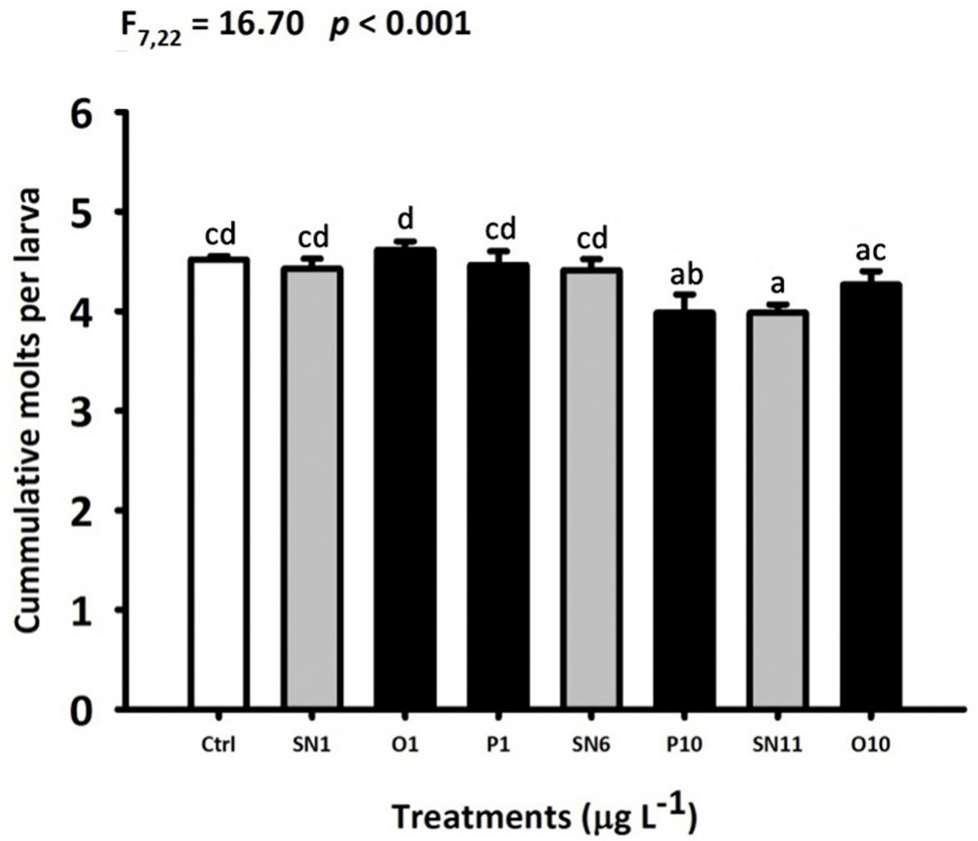
Effect of OAgNPs, PAgNPs and AgNO_3_ on the cumulative molts per larva of *B*. *amphitrite* on Day 5. Each bar represents the mean (+ SD) of four replicates. One-way ANOVA was used for the statistics analysis. Different letters above the bar indicate the means that are significantly different as identified by Tukey’s test. Ctrl: FSW control, SN: AgNO_3_, O: OAgNP and P: PAgNP.

The mean body length of barnacle larvae of the same development stage was not significantly different between treatments and control (*p* > 0.05 for all stages). Nevertheless, the 5-d old larvae from SN11, O10 and P10 treatments have less cumulative molts compared to the FSW control (F_7,22_ = 16.7; *p* < 0.001) (Fig 4).

**Fig 5:**
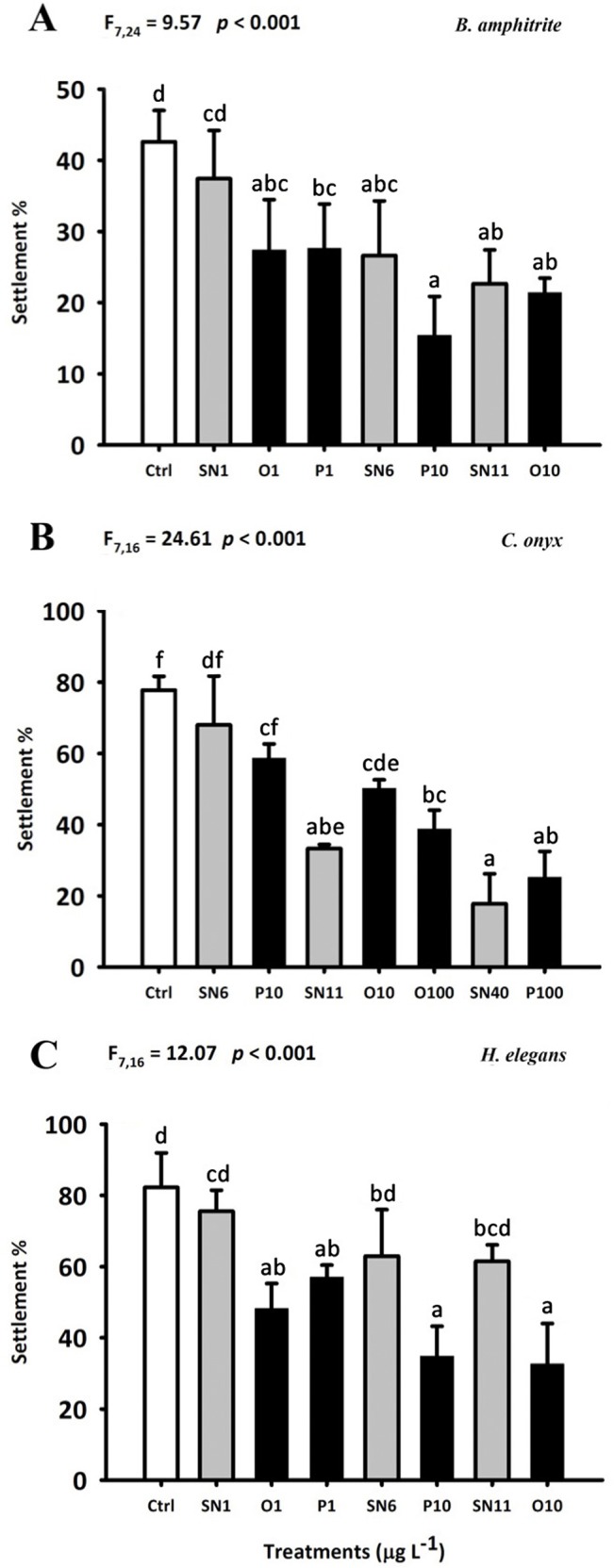
Effect of OAgNPs, PAgNPs and AgNO_3_ on the larval settlement of *B*. *amphitrite* (A), *C*. *onyx* (B) and *H*. *elegans* (C). Each bar represents the mean (+ SD) of four replicates (A) or three replicates (B-C). One-way ANOVA was used for the statistics analysis. Different letters above the bar indicate the means that are significantly different as identified by Tukey’s test. Ctrl: FSW control, SN: AgNO_3_, O: OAgNP and P: PAgNP.

The majority of larvae reached stage cyprid on day 5, and 15.4–42.6% of larvae settled and metamorphosed into barnacle juveniles (Fig 5). Significant reduction of percent settlement was observed in SN6, SN11, O1, O10, P1 and P10 treatments compared to the control (F_7,23_ = 9.57; *p* < 0.001). However, no significant difference in cumulative molts and percent settlement was found between SN1, O1 and P1 treatment; between SN6 and P10 treatments; and between SN11 and O10 treatments.

#### *C. onyx*

**Fig 6:**
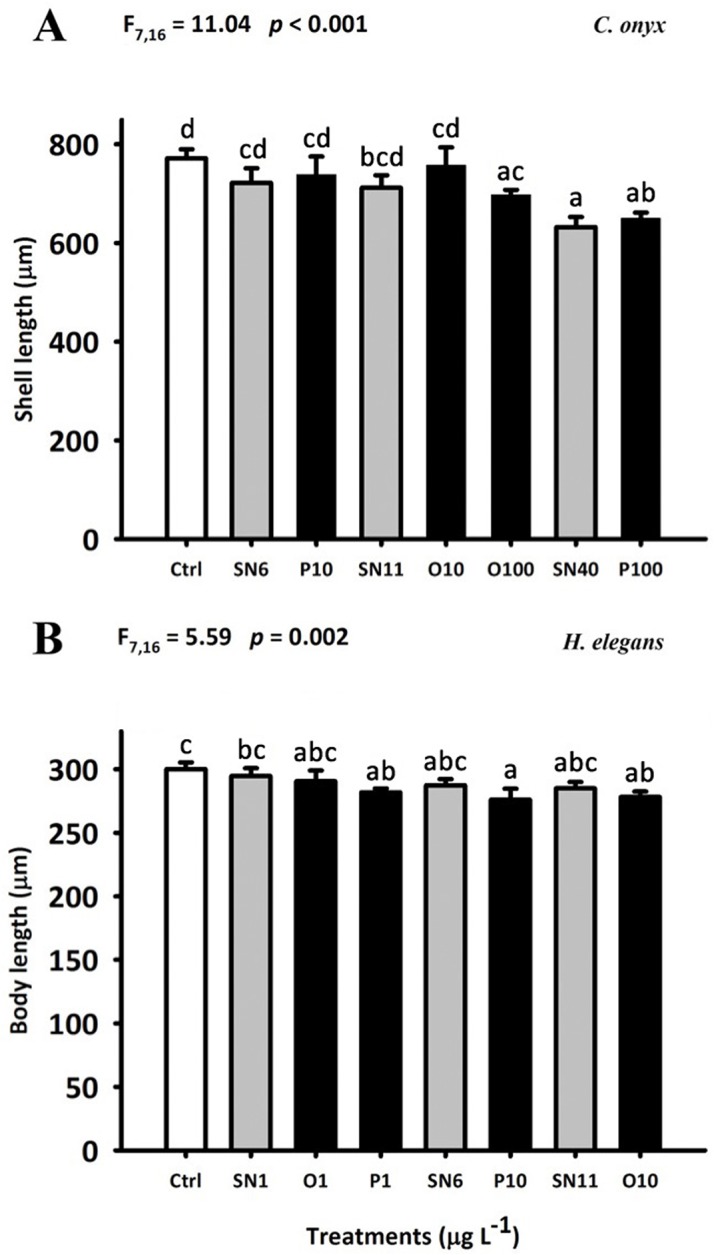
Effect of OAgNPs, PAgNPs and AgNO_3_ on the larval growth of *C*. *onyx* (A) and *H*. *elegans* (B). Each bar represents the mean (+ SD) of three replicates. One-way ANOVA was used for the statistics analysis. Different letters above the bar indicate the means that are significantly different as identified by Tukey’s test. Ctrl: FSW control, SN: AgNO_3_, O: OAgNP and P: PAgNP.

Significant reduction of the shell length of 8-d old *C*. *onyx* larvae was observed in SN40, O100 and P100 treatment (*p* < 0.05; Fig 6). *C*. *onyx* larvae became morphologically competent to metamorphose on day 8 (i.e. occurrence of shell brims), and 17.8–77.8% of them settled and metamorphosed in response to biofilms (Fig 5). All coated AgNPs and AgNO_3_ treatments, except SN6 and P10, significantly lowered the larval settlement rate (*p* < 0.05). Furthermore, no significant difference in larval growth and percent settlement was found between SN6 and P10; between SN11, O10 and O100 treatments; and between SN40 and P100 treatments.

#### *H. elegans*

The newly hatched larvae grew from a mean (± SD) body length of 73.4 ± 2.9 μm to a mean ranging from 276 ± 8 to 300 ± 5 μm during the 5-d chronic exposure. The treatments had significant effects on the body length of 5-d old larvae (F_7,15_ = 5.59; *p* = 0.002) (Fig 6). Nevertheless, there was no significant difference between coated AgNPs and the corresponding AgNO_3_ treatments (e.g. between SN1, O1 and P1). Most of the *H*. *elegans* larvae attained metamorphic competency at day 5 and the settlement rate ranged from 32.6 to 82.2% (Fig 5). Significant reduction of percent settlement was observed in O1, O10, P1 and P10 treatments compared to control. Furthermore, the settlement rate in the coated AgNPs treatment was significantly lower than that in the corresponding AgNO_3_ treatment (i.e. O1, P1 < SN1; P10 < SN6; O10 < SN11).

In all three larval species, different surface coating did not affect the toxicity of AgNPs on larval growth and percent settlement.

### Body burden of total silver

**Fig 7:**
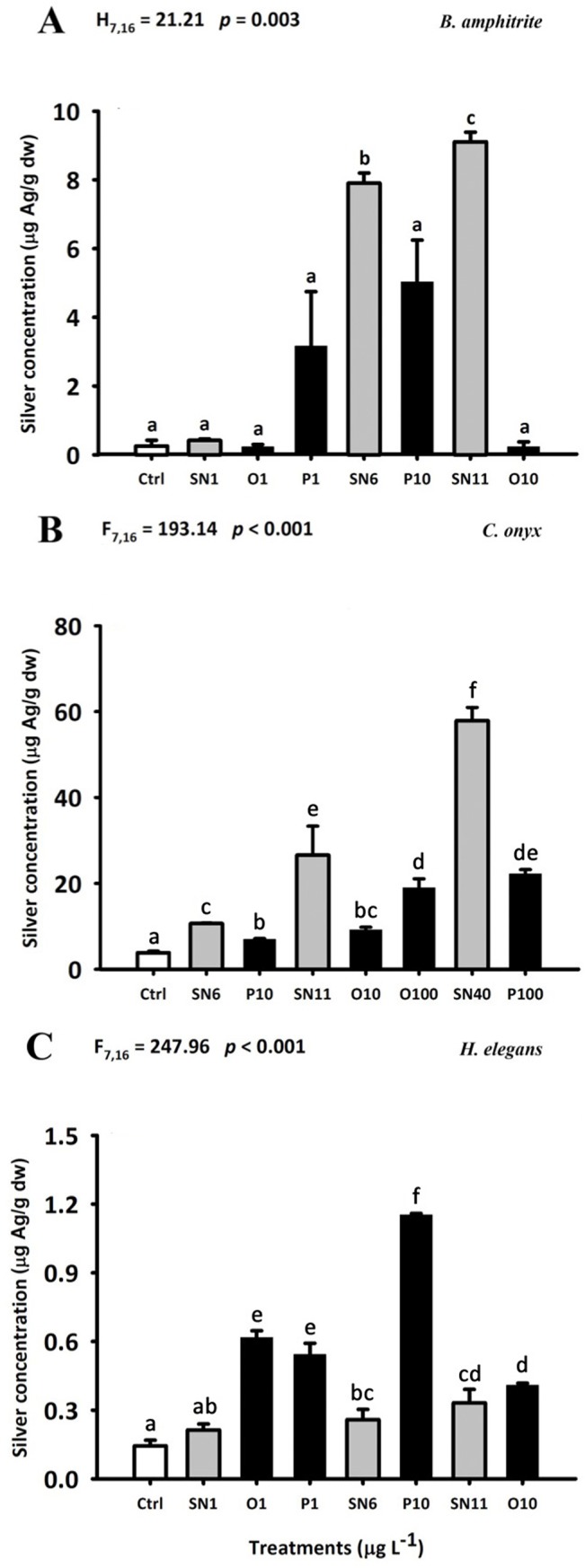
Body burden of total silver (μg g^-1^ dw) in larvae of *B*. *amphitrite* (A), *C*. *onyx* (B) and *H*. *elegans* (C). Each bar represents the mean (+ SD) of four replicates (A) or three replicates (B-C). In (B-C), One-way ANOVA was used for the statistics analysis. In (A), non-parametric Kruskal-Wallis One-way ANOVA on ranks was used for the statistical analysis. Different letters above the bar indicate the means that are significantly different as identified by Tukey’s test or Dunn’s test. Ctrl: FSW control, SN: AgNO_3_, O: OAgNP and P: PAgNP.

For *B*. *amphitrite*, a significant accumulation of silver was observed only in SN6 and SN11 treatments (Fig 7). The larvae in SN11 treatment accumulated the highest amount of silver (i.e. 9.12 μg g^-1^ dw).

For *C*. *onyx*, a significantly greater accumulation of silver was observed in all treatments compared to the control (Fig 7). The highest amount of accumulated silver (i.e. 57.9 μg g^-1^ dw) was observed in the larvae exposed to the SN40 treatment. The concentration of silver in larvae from AgNO_3_ treatments was significantly higher than that from the corresponding coated AgNP treatments (i.e. SN6 > P10; SN11 > O10, O100; SN40 > P100).

For *H*. *elegans*, there was a significantly greater accumulation of silver in all treatments, except SN1, compared to the control (Fig 7). The larvae in P10 treatment took up the highest amount of silver (i.e. 1.15 μg g^-1^ dw). Furthermore, while the concentration of silver accumulated in *H*. *elegans* from O1, P1 and P10 treatments was significantly higher than that from the corresponding SN1 and SN6 treatments (i.e. SN1 < O1, P1; SN6 < P10, the silver concentrations from SN11 and O10 treatments were not significantly different.

### Algal uptake of total silver

**Fig 8:**
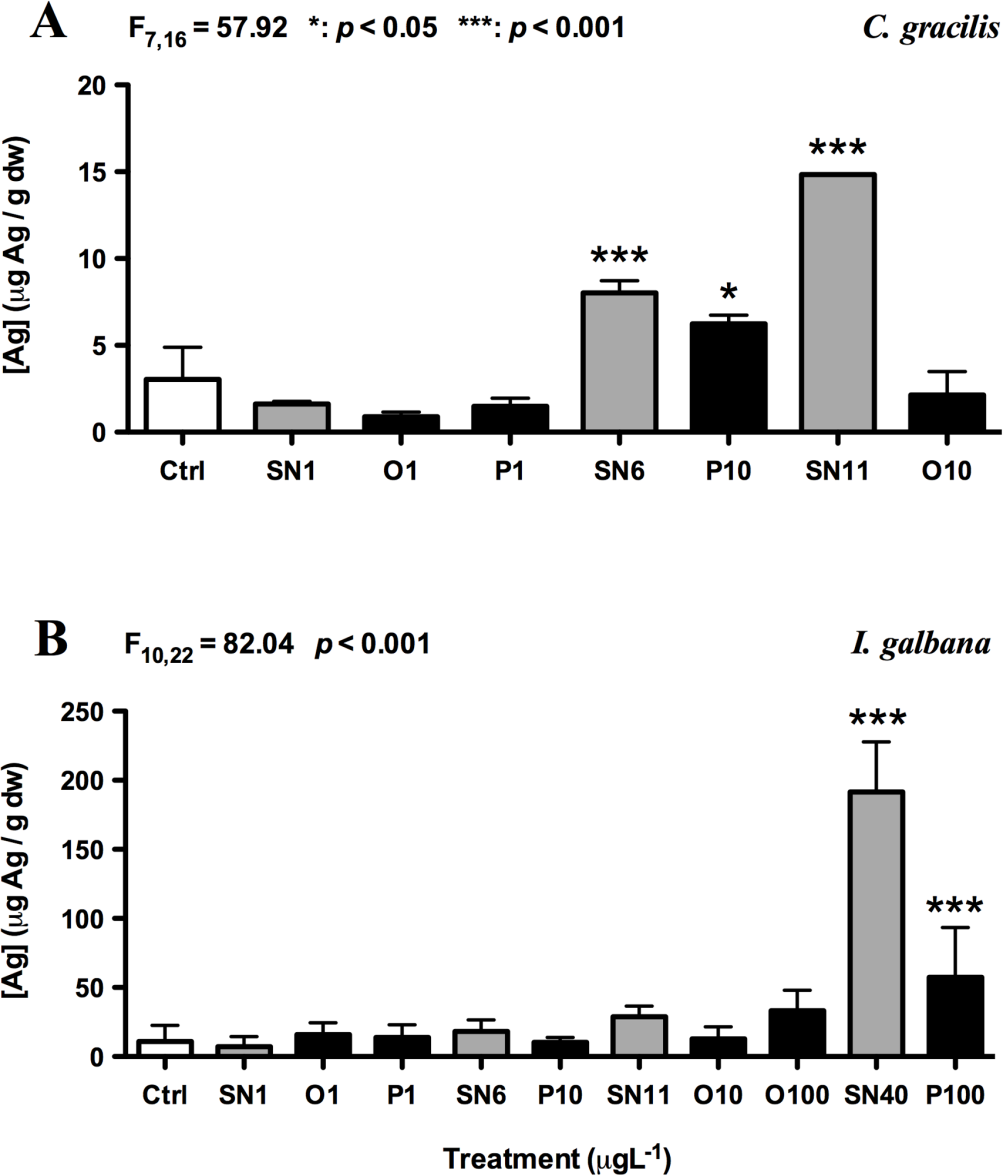
Total silver uptake (μg g^-1^ dw) by *C*. *gracilis* (A) and *I*. *galbana* (B) after 24 h of exposure. Each bar represents the mean (+ SD) of triplicates. One-way ANOVA was used for the statistics analysis. Significant different between FSW control and treatments was identified by Tukey’s test; *: *p* < 0.05; ***: *p* < 0.001. Ctrl: FSW control, SN: AgNO_3_, O: OAgNP and P: PAgNP.

After 24 h of exposure, a significant accumulation of Ag (*p* < 0.05) was observed in SN6-, SN11- and P10-treated *C*. *gracilis* and SN40- and P100-exposed *I*. *galbana* (Fig 8). The mean amount of Ag taken up in SN6-, SN11- and P10-treated *C*. *gracilis* was 8.02, 14.84 and 6.24 μg g^-1^ dw, respectively. *B*. *amphitrite*, which fed on *C*. *gracilis*, efficiently accumulated Ag from SN6 and SN11 treatment and the amount of Ag barnacle larvae took up after 5 d of exposure was slightly less than the algal uptake. Similarly, the amount of Ag taken up in SN40- and P100-exposed *I*. *galbana* was 191.5 and 57.3 μg g^-1^ dw after 24 h, which was also higher than the amount of Ag taken up by 8d-exposed *C*. *onyx* and 5d-exposed *H*.*elegans*. These results suggest that the Ag uptake rate in algae is higher than that in larvae.

### Biodistribution of coated AgNPs

**Fig 9:**
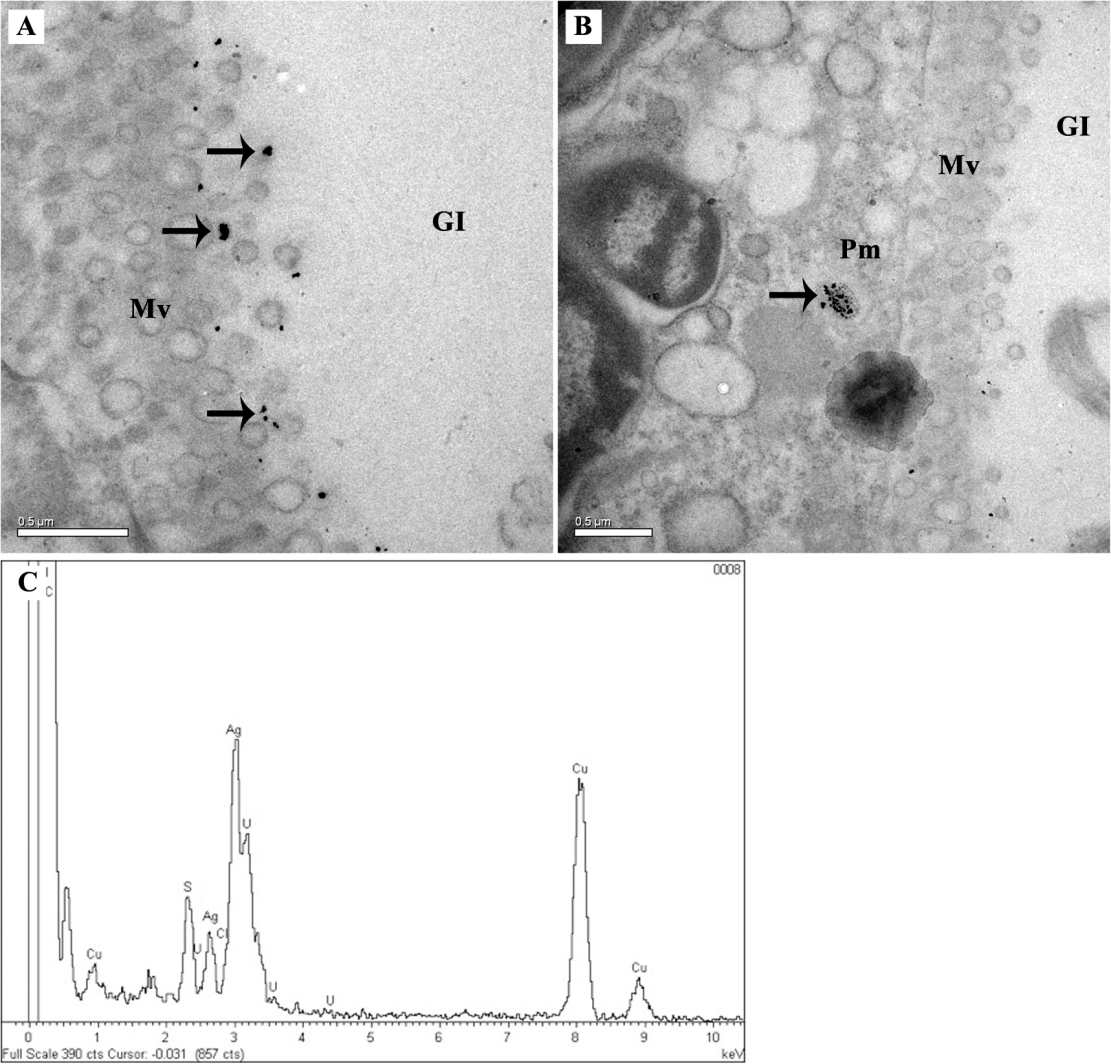
TEM micrographs of coated AgNP localization in *C*. *onyx* larva. Coated AgNPs were found in the intestine region of *C*. *onyx* treated with 100 μg L^**-1**^ of PAgNPs. Particles are visible in the intestine as black dots indicated by arrow. They are also observed to pass through the plasma membrane and deposit next to the microvilli (A) and in the vacuole (B). Gl: gut lumen; Mv: microvilli; Pm: plasma membrane. Scale bars: 0.5 μm. (C) is the EDS spectrum showing the presence of silver.

No silver was detected by EDS in *B*. *amphitrite* and *H*. *elegans* larvae. However, in the 100 μg L^-1^ PAgNP-exposed *C*. *onyx* larvae, coated AgNP aggregates were present in the gut lumen, next to the microvilli (Fig 9) and in the vacuoles of epithelial cell (Fig 9). The peaks on the EDS spectrum validated the presence of silver (Fig 9). The aggregates of coated AgNPs had broad size distribution ranging from 40–100 nm. In control and AgNO_3_-treated larvae, no silver was detected in the analytical spectrum of EDS in the intestine region.

## Discussion

In this proof of concept study, we have provided evidence that coated AgNPs adversely affected the larval stage of three different marine invertebrate species. Our results clearly indicate that chronic exposure to sub-lethal concentrations of coated AgNPs may lead to significant growth and development retardation, and reduction in settlement rates of the larvae *B*. *amphitrite*, *C*. *onyx* and *H*. *elegans* in a species-specific manner. The larvae are filter feeders and they can become susceptible to AgNPs when accumulating the particles through directly trapping them in mucus prior to ingestion or indirectly consuming microalgae with elevated Ag levels [5]. In particular, the filtration rate of *C*. *onyx* larvae could reach 0.4 ml hr^-1^ [41], leaving them highly vulnerable to the ingestion of AgNPs. For intertidal species, the recruitment success depends on at least five processes, including production of larvae, dispersal of larvae in plankton, larval settlement, and growth and survival of larvae and young juveniles [42]. While each dying individual must be replaced by one offspring recruited into the population if the population size is stable [43], the potential AgNPs toxicity on larval growth and settlement may result in changes in population dynamics and benthic community structure.

### Toxicity of coated AgNPs

Ag^+^ is a highly bioactive species and has been reported to possess a high toxicity to various species ranging from unicellular organisms to higher vertebrates in many laboratory toxicity experiments [32,44,45]. Ag^+^ also plays a major role in determining the toxicity of AgNPs, which undergo dissolution in most environmental conditions. Several authors have demonstrated that the toxicity of AgNPs, whether coated or naked, in marine organisms is dependent on the dissolved silver [15,16]. Cysteine, a strong Ag^+^ ligand, was used to determine the contribution of Ag^+^ to the toxicity of AgNPs. Results showed that the toxicity of uncoated AgNPs on the marine alga *Chattonella marina* decreased with an increasing concentration of cysteine [46]. Furthermore, Buffet et al. [28] demonstrated that uncoated AgNPs and Ag^+^ treated adult bivalve *Scrobicularia plana* showed similar oxidative-stress-related biomarker response. Opposite observation was made from a recent study showing that uncoated AgNPs were more toxic than their equivalent Ag^+^ dose and caused concentration dependent developmental defects in sea urchins [20]. Griffitt et al. [31] also revealed that chronic exposure of uncoated AgNPs induced significant adverse effects in both juvenile and adult sheepshead minnows and AgNPs were found to be more bioavailable than Ag^+^. The authors suggested that AgNP-driven toxicity was not attributable to the release of Ag^+^ through particle dissolution in the testing medium.

In the present study, a species-specific response to coated AgNPs has been clearly demonstrated. Both coated AgNPs and Ag^+^ reduced the larval growth rate of *C*. *onyx* as well as settlement rate in *B*. *amphitrite* and *C*. *onyx* by a similar extent (Figs 5 and 6), and silver was accumulated more readily in soluble form than coated AgNPs in *B*. *amphitrite* and *C*. *onyx* (Fig 7). In contrast, exposure to coated AgNPs reduced the growth (Fig 6) and larval settlement rate (Fig 5) in *H*. *elegans* and in most cases, coated AgNPs were more toxic than Ag^+^_._ Uptake rate of silver in coated AgNP-treated *H*. *elegans* was also higher than Ag^+^-treated counterparts, consistent with toxicity to larval settlement.

Ag is one of the common trace metals present in natural water and the dissolved form, Ag^+^, often associates with anionic ligands to achieve higher stability. In freshwater, the dominant silver species is the free toxic Ag^+^. With increasing [Cl^-^] (i.e. estuarine water), the dominant Ag species becomes the uncharged neutral chloride complex, AgCl_0(aq)_, which is highly bioavailable to biota. At high [Cl^-^] (i.e. seawater), the dominant species of inorganic Ag shifts to negatively charged silver chlorocomplexes (i.e. AgCl^2-^, AgCl_3_^2-^, AgCl_4_^3-^) which are less bioavailable than AgCl_0(aq)_. Early studies have demonstrated that, rather than Ag^+^, AgCl_0(aq)_ becomes the principal bioavailable species of inorganic silver with increasing [Cl^-^] (i.e. 0–50mM) [47,48] and may contribute to silver toxicity [49]. On the other hand, Groh et al. 2014 [50] has summarized that chloride has a profound protective effects on silver toxicity which is likely due to complexation and precipitation of most of the free Ag^+^. Therefore, definite conclusion on the effect of chloride on silver toxicity is impossible unless more complete information concerning silver speciation is provided.

In this study, coated AgNPs were directly added to the culture medium and diet was not the intended exposure route. Given that *C*. *gracilis* and *I*. *galbana* could accumulate Ag in higher concentrations of AgNO_3_ and PAgNP treatments (Fig 8), it could not be excluded that, in those tested concentrations, dietary effect might exist. A number of studies have previously shown that algae as a vector of Ag transfer could alter the bioavailability and toxicity of coated and naked AgNPs [9,28,51].

### Biodistribution of coated AgNPs

Previous *in vitro* studies have shown that AgNPs were introduced in the cytoplasm of human hepatoma cells, rat primary neural cells, and rainbow trout gill cells [52–54], and this was further supported by *in vivo* studies in terrestrial plant, freshwater algae and fish [55–57]. In our observation under TEM, coated AgNPs appeared in aggregates at the gut lumen of the *C*. *onyx* larvae (Fig 9). This observation was in agreement with earlier studies on naked AgNPs which showed that arthropods and mollusks concentrated silver nano-particulates in digestive organs [58,59]. As depicted in the Fig 8, the sizes of aggregates of coated AgNPs were averagely smaller (i.e. 40–100 nm) than that of the aggregates in FSW (i.e. 920 nm at 100 μg L^-1^ of PAgNP). According to Ward et al. [60,61], in filter feeders, food aggregates are likely to be broken down by the action of cilia on the feeding apparatus and gills. So, it is possible that the large aggregates of AgNPs can be broken down into smaller ones and subsequently, pass through the plasma membrane via endocytosis. Endocytosis has been proposed to be the general route of uptake of particles or molecules up to 100 nm in size in digestive system [62]. The endocytotic process internalizes particles by invagination of the lipid plasma membrane, formation of vesicles that enclose the particles and transportation of vacuoles into the cell [1]. In the present study, aggregates of coated AgNPs were observed to pass across the plasma membrane and be taken up in the vacuoles in epithelial cells (Fig 9). Likewise, it was demonstrated that citrate coated AgNPs were transported across the cell membrane and internalized inside the vacuoles of the polychaete *Nereis diversicolor* [63]. Vacuoles serve as lytic organelles and storage compartments of inorganic ions and organic compounds [64]. Their predominant role in heavy metal cation sequestration and detoxication in cells of marine invertebrates is widely recognized [65]. Nevertheless, caution should be exercised when gauging the AgNPs behavior in larval digestive system with distinct pH environment. Stumpp et al. (2013) have suggested that the stomach pH of sea urchin pluteus larvae ranges between pH 8.9 to pH 9.6 [66].

### Influence of coating materials on toxicity of AgNPs

Silver is not thermodynamically stable under most of the environmental conditions, so molecules like citrate, carboxylic acids, polymers or polysaccharides are applied as surfactants to stabilize AgNPs. Although the coating materials showed no biological effect [23,67], it has been reported that due to different physiochemical properties, they play essential roles on NP toxicity and/or their mode of action upon release to the environment [68]. In addition, hydrophilicity has been considered as one of the major factors governing NP toxicity. Yang et al. [27] have systemically investigated the effect of coatings on the toxicity of AgNPs on *C*. *elegans*, and found that AgNPs coated with gum arabic (GA) (relatively hydrophilic) was about 9-fold more toxic than PAgNPs (relatively hydrophobic). Since the two types of coated AgNPs had a similar particle size, it was suggested that the difference in toxicity was attributed to the type of surface coating and silver dissolution rate. As GA provided a more hydrophilic surface for coated AgNPs, it reduced the formation of aggregates, increased the available surface area for dissolution, and hence, harbored a higher toxicity. On the other hand, there is experimental evidence that hydrophobic coated AgNPs could exert a higher toxicity as hydrophobic polymer has a greater affinity to plasma membrane and promotes the transport and penetration of coated AgNPs via the peptidoglycan layer, causing the disruption of cell membrane, release of cytoplasmic constituents and cell death [69].

In our chronic toxicity test, despite PAgNPs having a smaller aggregation size and releasing about similar amount of (at 10 μg L^-1^) or 4-fold more Ag^+^ (at 100 μg L^-1^) than OAgNPs, there was no observable difference in their effects on the marine invertebrate larvae. This result agreed with the earlier findings, in which no significant difference could be found between the toxicity of OAgNPs and PAgNPs on the survivorship and reproduction of the earthworm *Eisenia fetida* [70]. The same study also showed that coatings themselves (i.e. oleic acid and polyvinylpyrrolidone) did not exhibit any toxicity to the organisms. Although self-aggregation of NPs into larger particle masses would hinder the release of its ions, it would increase the bioavailability in suspension feeding [71]. Ward and Kach [70] demonstrated that suspension-feeder bivalves captured particles larger than 6 μm with 90% efficiency, and the capture efficiency decreased with decreasing particle size. Thus, effect of hydrophilicity on NP toxicity may not be solely due to the ion release efficiency.

## References

[1] Luoma SN. Silver nanotechnologies and the environment: Old problem or new challenges? Project on Emerging Nanotechnologies. Available: http://www.nanotechproject.org/process/assets/files/7036/nano_pen_15_final.pdf. Accessed 26 November 2011.

[2] Woodro Wilson International Center. Project on Emerging Nanotechnologies. 2011. Available: http://www.nanotechproject.org/index.php?id=44&action=view. Accessed 13 January 2012.

[3] Klaine SJ, Alvarez RJJ, Batley GE, Fernandes TF, Handy RD, Lyon DY, et al Nanomaterials in the environment: Behavior, fate, bioavailability, and effects. Environ Toxicol Chem. 2008;27: 1825–1851. 19086204 10.1897/08-090.1

[4] US Environmental Protection Agency. Nanotechnology white paper. USEPA; 2007. Available: http://www.epa.gov/osainter/pdfs/nanotech/epa-nanotechnology-whitepaper-0207.pdf. Accessed 21 February 2012.

[5] Baker TJ, Tyler CR, Galloway TS. Impacts of metal and metal oxide nanoparticles on marine organisms. Environ Pollut. 2014;186: 257–271. 10.1016/j.envpol.2013.11.014 24359692

[6] Navarro E, Piccapietra F, Wagner B, Marconi F, Kaegi R, Odzak N, et al Toxicity of silver nanoparticles to *Clamydomonas reinhardtii* . Environ Sci Technol. 2008;42: 8959–8964. 19192825 10.1021/es801785m

[7] Gao J, Youn S, Hovsepyan A, Llaneza VL, Wang Y, Bitton G, et al Dispersion and toxicity of selected manufactured nanomaterials in natural river water samples: Effects of water chemical composition. Environ Sci Technol. 2009;43: 3322–3328. 19534153 10.1021/es803315v

[8] Kvitek L, Vanickova M, Panacek A, Soukupova J, Dittrich M, Valentova E, et al Initial study on the toxicity of silver nanoparticles (NPs) against *Paramecium caudatum* . J Phys Chem C. 2009;113: 4296–4300.

[9] Croteau MNI, Misra SK, Luoma SN, Valsami-Jones E. Silver bioaccumulation dynamics in a freshwater invertebrate after aqueous and dietary exposures to nanosized and ionic Ag. Environ Sci Technol 2011;45: 6600–6607. 10.1021/es200880c 21667957

[10] Kim KT, Truong L, Wehmas L, Tanguay RL. Silver nanoparticle toxicity in the embryonic zebrafish is governed by particle dispersion and ionic environment. Nanotechnology 2013;24(11): 115101 10.1088/0957-4484/24/11/115101 23449170 PMC3782284

[11] French RA, Jacobson AR, Kim B, Isley SL, Penn RL, Baveye PC. Influence of ionic strength, pH, and cation valence on aggregation kinetics of titanium dioxide nanoparticles. Environ Sci Technol. 2009;43: 1354–1359. 19350903 10.1021/es802628n

[12] Wong SWY, Leung PTY, Djurišić AB, Leung KMY. Toxicities of nano zinc oxide to five marine organisms: Influences of aggregate size and ion solubility. Anal Bioanal Chem. 2010;396: 609–618. 10.1007/s00216-009-3249-z 19902187

[13] Quik JTK, Vonk JA, Hansen SF, Baun A, Van De Meent D. How to assess exposure of aquatic organisms to manufactured nanoparticles? Environ Int. 2011;37: 1068–1077. 10.1016/j.envint.2011.01.015 21411153

[14] Burchardt AD, Carvalho RN, Valente A, Nativo P, Gilliland D, Garcia CP, et al Effects of silver nanoparticles in diatom *Thalassiosira pseudonana* and *Cyanobacterium Synechococcus sp* . Environ Sci Technol. 2012;46: 11336–11344. 10.1021/es300989e 22958173

[15] Macken A, Byrne HJ, Thomas KV. Effects of salinity on the toxicity of ionic silver and Ag-PVP nanoparticles to *Tisbe battagliai* and *Ceramium tenuicorne* . Ecotoxicol Environ Saf. 2012;86: 101–110. 10.1016/j.ecoenv.2012.08.025 23036305

[16] Turner A, Brice D, Brown MT. Interactions of silver nanoparticles with the marine macroalga, *Ulva lactuca* . Ecotoxicology 2012;21: 148–154. 10.1007/s10646-011-0774-2 21877230

[17] Gomes T, Pereira CG, Cardoso C, Bebianno MJ. Differential protein expression in mussels *Mytilus galloprovincialis* exposed to nano and ionic Ag. Aquat Toxicol. 2013;136–137: 79–90. 10.1016/j.aquatox.2013.03.021 23665239

[18] McCarthy MP, Carroll DL, Ringwood AH. Tissue specific responses of oysters, *Crassostrea virginica*, to silver nanoparticles. Aquat Toxicol. 2013;138–139: 123–128. 10.1016/j.aquatox.2013.04.015 23728357

[19] Ringwood AH, McCarthy M, Bates TC, Carroll DL. The effects of silver nanoparticles on oyster embryos. Mar Environ Res. 2010;69 Supp1: S49–S51. 19913905 10.1016/j.marenvres.2009.10.011

[20] Siller L, Lemloh ML, Piticharoenphun S, Mendis BG, Horrocks BR, Brümmer F, et al Silver nanoparticle toxicity in sea urchin *Paracentrotus lividus* . Environ Pollut. 2013;178: 498–502. 10.1016/j.envpol.2013.03.010 23561841

[21] Pechenik JA. On the advantages and disadvantages of larval stages in benthic marine invertebrate life cycles. Mar Ecol Prog Ser. 1999;177: 269–297.

[22] García-Alonso J, Rodriguez-Sanchez N, Misra SK, Valsami-Jones E, Croteau MN, Luoma SN, et al Toxicity and accumulation of silver nanoparticles during development of the marine polychaete *Platynereis dumerili* . Sci Total Environ. 2014;476: 688–695. 10.1016/j.scitotenv.2014.01.039 24514586

[23] Kwok KWH, Auffan M, Badireddy AR, Nelson CM, Wiesner MR, Chilkoti A, et al Uptake of silver nanoparticles and toxicity to early life stages of Japanese medaka (*Oryzias latipes*): Effect of coating materials. Aquat Toxicol. 2012;120–121: 59–66. 10.1016/j.aquatox.2012.04.012 22634717

[24] Meyer JN, Lord CA, Yang XY, Turner EA, Badireddy AR, Marinakos SM, et al Intracellular uptake and associated toxicity of silver nanoparticles in *Caenorhabditis elegans* . Aquat Toxicol. 2010;100: 140–150. 10.1016/j.aquatox.2010.07.016 20708279

[25] Powers CM, Slotkin TA, Seidler FJ, Badireddy AR, Padilla S. Silver nanoparticles alter zebrafish development and larval behavior: distinct roles for particle size, coating and composition. Neurotoxicol Teratol. 2011;33: 708–714. 10.1016/j.ntt.2011.02.002 21315816 PMC3112298

[26] Fabrega J, Luoma SN, Tyler CR, Galloway TS, Lead JR. Silver nanoparticles: behaviour and effects in the aquatic environment. Environ Int. 2011;37: 517–531. 10.1016/j.envint.2010.10.012 21159383

[27] Yang X, Gondikas AP, Marinakos SM, Auffan M, Liu J, Hsu-Kim H, et al Mechanism of silver nanoparticle toxicity is dependent on dissolved silver and surface coating in *Caenorhabditis elegans* . Environ Sci Technol. 2012;46: 1119–1127. 10.1021/es202417t 22148238

[28] Buffet PE, Pan JF, Poirier L, Amiard-Triquet C, Amiard J, Gaudin P, et al Biochemical and behavioural responses of the endobenthic bivalve *Scrobicularia plana* to silver nanoparticles in seawater and microalgal food. Ecotoxicol Environ Saf. 2013;89: 117–124. 10.1016/j.ecoenv.2012.11.019 23260182

[29] Hwang ET, Lee JH, Chae YJ, Kim YS, Kim BC, Sang BI, et al Analysis of the toxic mode of action of silver nanoparticles using stress-specific bioluminescent bacteria. Small 2008;4(6): 765–750. 10.1002/smll.200700954 18528852

[30] Choi O, Hu Z. Size dependent and reactive oxygen species related nanosilver toxicity to nitrifying bacteria. Environ Sci Technol. 2008;42: 4583–4588. 18605590 10.1021/es703238h

[31] Griffitt RJ, Brown-Peterson NJ, Savin DA, Manning CS, Boube I, Ryan RA, et al Effects of chronic nanoparticulate silver exposure to adult and juvenile sheepshead minnows (*Cyprinodon variegates*). Environ Toxicol Chem. 2012;30: 160–167. 10.1002/etc.709 21994144

[32] Fabrega J, Fawcett SR, Renshaw JC, Lead JR. Silver nanoparticle impact on bacterial growth: effect of pH, concentration, and organic matter. Environ Sci Technol. 2009;43: 7285–7290. 19848135 10.1021/es803259g

[33] US Environmental Protection Agency. SW-846 reference methodology: Method 3010A: Acid digestion of aqueous samples and extracts for total metals for analysis by FLAA or ICP spectroscopy USEPA; 1992 Available: http://www.epa.gov/osw/hazard/testmethods/sw846/pdfs/3010a.pdf. Accessed 12 March 2012.

[34] Shikuma NJ, Hadfield MG. Temporal variation of an initial marine biofilm community and its effects on larval settlement and metamorphosis of the tubeworm *Hydroides elegans* . Biofilms 2005;2: 231–238.

[35] Chiu JMY, Zhang R, Wang H, Thiyagarajan V, Qian PY. Nutrient effects on intertidal community: from bacteria to invertebrates. Mar Ecol Prog Ser. 2008;358: 41–50.

[36] Qiu JW, Qian PY. Effects of food availability, larval source and culture method on larval development of *Balanus amphitrite* amphitrite Darwin: implications for experimental design. J Exp Mar Biol Ecol. 1997;217: 47–61.

[37] Chiu JMY, Po BHK, Chan CYS, Lam MHW, Qian PY, Wu RSS. Polybrominated diphenyl ethers (PBDEs) alter larval settlement of marine intertidal organisms across three phyla via reducing bacterial abundance on the biofilms. Environ Sci Technol. 2012;46: 7772–7781. 10.1021/es300261c 22697365

[38] Guillard RLR. Culture of phytoplankton for feeding marine invertebrates In: Walter SL, Matoira CH, editors. Culture of Marine Invertebrate Animals. New York: Springer; 1975 pp. 29–60.

[39] Zar JH. Biostatistical analysis, 4th ed. Englewood Cliffs, NJ: Prentice Hall; 1999. 944 p.

[40] Shapiro SS, Wilk MB. An analysis of variance test for normality (complete samples). Biometrics 1965;52: 59–611.

[41] Chiu JMY, Ng TYT, Wang WX, Thiyagarajan V, Qian PY. Latent effects of larval food limitation on filtration rate, carbon assimilation and growth in juvenile gastropod *Crepidula onyx* . Mar Ecol Prog Ser. 2007;343: 173–182.

[42] Underwood AJ, Keough MJ. Supply-side ecology–the nature and consequences of variations in recruitment of intertidal organisms In: Bertness MD, Gaines SD, Hay ME, editors. Marine Community Ecology. Sunderland, MA: Sinauer Associates; 2001 pp. 183–200.

[43] Menge BA, Branch GM. Rocky intertidal communities In: Bertness MD, Gaines SD, Hay ME, editors. Marine Community Ecology. Sunderland, MA: Sinauer Associates; 2001 pp. 221–252.

[44] Miao AJ, Schwehr KA, Xu C, Zhang SJ, Luo Z, Quigg A, et al The algal toxicity of silver engineered nanoparticles and detoxification by exopolymeric substances. Environ Pollut. 2009;157: 3034–3041. 10.1016/j.envpol.2009.05.047 19560243

[45] Oukarroum A, Barhoumi L, Pirastru L, Dewez D. Silver nanoparticle toxicity effect on growth and cellular viability of the aquatic plant *Lemna gibba* . Environ Toxicol Chem. 2013;32: 902–907. 10.1002/etc.2131 23341248

[46] He D, Dorantes-Aranda JJ, Waite TD. Silver nanoparticle–Algae interactions: Oxidative dissolution, reactive oxygen species generation and synergistic toxic effects. Enviro Sci Technol 2012;46: 8731–8738. 10.1021/es300588a 22816991

[47] Engel DW, Sunda WG, Fowler BA. Factors affecting trace metal uptake and toxicity to estuarine organisms. I. Environmental parameters In: Vernberg FJ, Calabrese A, Thurberg FP, Vernberg WB, editors. Biological Monitoring of Marine Pollutions. Waltham, MA: Academic; 1981 pp: 127–144.

[48] Reinfelder JR, Chang SI. Speciation and microalgal bioavailability of inorganic silver. Environ Sci Technol 1999;33: 1860–1863.

[49] Erickson RJ, Brooke LT, Kahl MD, Venter FV, Harting SL, Markee TP, et al Effects of laboratory test conditions on the toxicity of silver to aquatic organisms. Environ Toxicol Chem. 1998;17: 572–578.

[50] Groh KJ, Dalkvist T, Piccapietra F, Behra R, Suter MJF, Schirmer K. Critical influence of chloride ions on silver ion-mediated acute toxicity of silver nanoparticles to zebrafish embryos. Nanotoxicology 2015;9: 81–91. 10.3109/17435390.2014.893379 24625062

[51] Brix KV, Gillette P, Pourmand A, Capo TR, Grosell M. The effects of dietary silver on larval growth in the echinoderm *Lytechinus variegatus* . Arch Environ Contam Toxicol. 2012;63: 95–100. 10.1007/s00244-012-9757-4 22434452

[52] Farkas J, Christian P, Gallego-Urrea JA, Roos N, Hassellöv M, Tollefsen KE, et al Uptake and effects of manufactured silver nanoparticles in rainbow trout (*Oncorhynchus mykiss*) gill cells. Aquat Toxicol. 2011;101: 117–125. 10.1016/j.aquatox.2010.09.010 20952077

[53] Haase A, Rott S, Mantion A, Graf P, Plendl J, Thünemann AF, et al Effects of silver nanoparticles on primary mixed neuronal cell cultures: Uptake, oxidative stress and acute calcium response. Toxicol Sci. 2012;126: 457–468. 10.1093/toxsci/kfs003 22240980 PMC3307608

[54] Yu SJ, Chao JB, Su J, Yin YG, Liu JF, Jiang GB. Quantification of the uptake of silver nanoparticles and ions to HepG2 cells. Environ Sci Technol. 2013;47: 3268–3274. 10.1021/es304346p 23458171

[55] Ma X, Geiser-Lee J, Deng Y, Kolmakov A. Interactions between engineered nanoparticles (ENPs) and plants: Phytotoxicity, uptake and accumulation. Sci Total Environ. 2010;408: 3053–3061. 10.1016/j.scitotenv.2010.03.031 20435342

[56] Miao AJ, Luo Z, Chen CS, Chin WC, Santschi PH, Quigg A. Intracellular uptake: A possible mechanism for silver engineered nanoparticle toxicity to a freshwater alga *Ochromonas danica* . PLOS ONE 2010;5(12): e15196 10.1371/journal.pone.0015196 21203552 PMC3008680

[57] Scown T, Santos E, Johnston B, Gaiser B, Baalousha M, Mitov S, et al Effects of aqueous exposure to silver nanoparticles of different sizes in rainbow trout. Toxicol Sci. 2010;115: 521–534. 10.1093/toxsci/kfq076 20219766

[58] Zuykov M, Pelletier E, Demers S. Colloidal complexed silver and silver nanoparticles in extrapallial fluid of *Mytilus edulis* . Mar Environ Res. 2011;71: 17–21. 10.1016/j.marenvres.2010.09.004 20950850

[59] Al-Sid-Cheikh M, Rouleau C, Pelletier E. Tissue distribution and kinetics of dissolved and nanoparticulate silver in Iceland scallop (*Chlamys islandica*). Mar Environ Res. 2013;86: 21–28. 10.1016/j.marenvres.2013.02.003 23489838

[60] Ward JE, MacDonald BA, Thompson RJ, Beninger PG. Mechanisms of suspension-feeding in bivalves: resolution of current controversies by means of endoscopy. Limnol Oceanogr. 1993;38: 265–272.

[61] Ward JE, Newell RIE, Thompson RJ, MacDonald BA. In vivo studies of suspension-feeding processes in the oyster *Crassostrea virginica* (Gmelin). Biol Bull. 1994;186: 221–240. 29281372 10.2307/1542056

[62] Iversen TG, Skotland T, Sandvig K. Endocytosis and intracellular transport of nanoparticles: Present knowledge and need for future studies. Nanotoday 2011;6: 176–185.

[63] García-Alogso J, Khan FR, Misra SK, Turmaine M, Smith BD, Rainbow PS, et al Cellular internalization of silver nanoparticles in gut epithelia of the estuarine polychaete *Nereis diversicolor* . Environ Sci Technol. 2011;45: 4630–4636. 10.1021/es2005122 21517067

[64] Matile P. Biochemistry and function of vacuoles. Ann Rev Plant Physiol. 1978;29: 193–213.

[65] Ahearn GA, Mandal PK, Mandal A. Mechanisms of heavy-metal sequestration and detoxification in crustaceans: a review. J Comp Physiol B. 2004;174: 439–452. 15243714 10.1007/s00360-004-0438-0

[66] Stumpp M, Hu M, Casties I, Saborowski R, Bleich M, Meizner F, et al Digestion in sea urchin larvae impaired under ocean acidification. Nat Clim Change. 2013;3: 1044–1049.

[67] Shoults-Wilson WA, Reinsch BC, Tsyusko O, Bertsch PM, Lowry GV, Unrine JM. Effect of silver nanoparticle surface coating on bioaccumulation and reproductive toxicity in earthworms (*Eisenia fetida*). Nanotoxicology 2011;5: 432–444. 10.3109/17435390.2010.537382 21142839

[68] Farré M, Gajda-Schrantz M, Kantiani L, Barceló D. Ecotoxicity and analysis of nanomaterials in the aquatic environment. Anal Bioanal Chem. 2009;393: 81 **–** 95. 10.1007/s00216-008-2458-1 18987850

[69] Dutta S, Shome A, Kar T, Das PK. Counterion-induced modulation in the antimicrobial activity and biocompatibility of amphiphilic hydrogelators: influence of in-situ-synthesized Ag nanoparticle on the bactericidal property. Langmuir 2011;27: 5000–5008. 10.1021/la104903z 21446701

[70] Ward JE, Kach DJ. Marine aggregates facilitate ingestion of nanoparticles by suspension-feeding bivalves. Mar Environ Res. 2009;68: 137–142. 10.1016/j.marenvres.2009.05.002 19525006

[71] Canesi L, Ciacci C, Fabbri R, Marcomini A, Pojana G, Gallo G. Bivalve mollusks as a unique target group for nanoparticle toxicity. Mar Environ Res. 2012;76: 16–21. 10.1016/j.marenvres.2011.06.005 21767873

